# Clinical and Demographic Determinants of Adherence to Digital Monitoring in Cirrhosis: A Prospective Cohort Analysis

**DOI:** 10.21203/rs.3.rs-9633932/v1

**Published:** 2026-05-19

**Authors:** Adam P Buckholz, Avesh Thuluvath, Kamaryn Tanner, Brian McSteen, Olivia Blocker, Ana C. Krieger, Alan A. Cohen, Elizabeth C. Verna, Robert S. Brown

**Affiliations:** NewYork-Presbyterian/Weill Cornell Medical Center; NewYork-Presbyterian/Columbia University Medical Center; Columbia University Mailman School of Public Health; NewYork-Presbyterian/Weill Cornell Medical Center; NewYork-Presbyterian/Weill Cornell Medical Center; NewYork-Presbyterian/Weill Cornell Medical Center; Columbia University Mailman School of Public Health; NewYork-Presbyterian/Columbia University Medical Center; NewYork-Presbyterian/Weill Cornell Medical Center

**Keywords:** Data Accuracy, Wearable Electronic Devices, Liver Cirrhosis, Early Diagnosis, Cognition, Hepatic Encephalopathy, Sociodemographic Factors

## Abstract

**Background:**

Cirrhosis has a high burden of care and frequent hospitalizations. Remote monitoring with digital wearable technology may improve early diagnosis and intervention with passive, convenient data collection and transmission, but its usability has not been systematically assessed in cirrhosis. We evaluate the factors associated with adherence to longitudinal monitoring in a diverse, prospective study of patients with cirrhosis.

**Methods:**

Patients with cirrhosis were prospectively enrolled from 2023–2025. Baseline clinical, laboratory, survey and cognitive data were collected, and each participant provided with an Oura 3.0 ring. Adherence was defined as the frequency of days with at least 80% of the day with wearable data transmission. Additional factors related to adherence, including data quality, dropout and attrition were assessed. Associations between clinical and sociodemographic variables and likelihood of adherence were assessed in mixed effects models.

**Results:**

Of 111 participants were enrolled, at least 80% data transmission occurred in 65.6% of eligible days. Across the study period, collected days had high data completeness (> 95%). Sociodemographic factors were not associated with adherence, but patients with decompensated cirrhosis or Child-Turcotte-Pugh B/C had lower adherence. In adjusted analysis, impaired cognition (OR 0.26 [95% CI 0.10, 0.68]) or a history of hepatic encephalopathy (HE) (OR 0.30 [95% CI 0.13, 0.71]) at baseline were the strongest predictors of reduced adherence.

**Discussion:**

We observed high adherence to wearable fitness tracking in cirrhosis, with significant within-day data quality, suggesting feasibility of remote monitoring. Contrary to expectation, severity of liver disease, especially cognition/HE, predicted adherence, not sociodemographics. While wearables are likely readily feasible in compensated cirrhosis patients for detecting progression, making remote monitoring successful in high-risk patients will require strategies to facilitate engagement and retention.

## Background

Cirrhosis is a common and highly morbid condition affecting more than two million people in the United States.^1^ Annually, approximately 5–7% of patients living with cirrhosis will suffer a new decompensating complication, including variceal hemorrhage, hepatic encephalopathy or abdominal ascites.^2^ Those with compensated cirrhosis have an annual mortality of approximately 1–5%, compared to 50–100% after decompensation.^3^ A recent longitudinal analysis found that the cost of cirrhosis care more than doubles ($47,631 vs $20,996) among those who develop a decompensation, higher than the cost of heart failure and chronic obstructive pulmonary disease.^4^ In this study, hospitalization was the greatest driver of increased cost, with decompensated patients have re-admission rates up to 50% within three months of hospital discharge.^5^ Moreover, many of these hospitalizations and re-hospitalizations were potentially avoidable if the inciting condition, such as infection, worsening ascites, or progressive encephalopathy, were detected and managed in the ambulatory setting. Unfortunately, many patients with cirrhosis are unable to access timely outpatient care due to lack of access to specialist care,^6^ increased burden on caregivers, low health literacy, or financial constraints. Less than half of patients receive post-discharge follow up within 90 days,^7^ leading to infrequent observation and contributing to readmission, expense, and poor outcomes. Taken together, clinicians lack adequate detection and monitoring tools for cirrhosis and its complications, leading to expensive and deadly events.

Digital health technology^8^ may be able to help bridge some of the care gap in cirrhosis, reducing hospitalizations, improving outcomes and patient wellbeing.^9^ Smartphones have become nearly ubiquitous in the United States, with over 85% of the population possessing a smartphone and 31% possessing fitness trackers, by one estimate.^10^ Among the most promising potential applications of digital technology in liver disease is the use of wearable technology for remote monitoring. Consumer-grade wearables, hereafter “wearables”, are electronic devices using sensors and embedded software to collect and interpret a wide array of health metrics including physical activity, heart rate, respiratory rate and blood pressure. Several studies have suggested that digital biomarkers may be able to predict important complications of liver disease, including hepatic encephalopathy (HE)^11,12^ and worsening ascites.^13^ Digital biomarkers could allow real-time clinical prediction and remote management, and there is some evidence that collection of remote data can improve patient access to healthcare,^14^ provide data reflective of how disease affects patients day-to-day (not captured in data from controlled healthcare settings),^15^ and improve outcomes.^16^

There are, however, several possible barriers to large scale implementation in healthcare generally,^17^ and there are currently no clinical care pathways that incorporate wearable technology or remote monitoring for disease management in cirrhosis. Most studies evaluating the use of remote monitoring/wearable technology are in healthy volunteers and use in liver disease is generally restricted to pilot studies of small populations in controlled settings. Additionally, patients with cirrhosis may be less likely to have access to smart phones, which are critical for data transmission. One study from 2019 found that approximately 72% of patients with decompensated cirrhosis possessed a smartphone.^18^ These patients were younger, more likely to be working, had higher education status and higher mean household income. Likewise, usefulness of remote monitoring and intervention using wearables depends on longitudinal adherence to the monitoring strategy. One nationwide study evaluating patients both with and without a chronic disease found that African American and Hispanic/Latino patients had lower rates of retention, while older patients had higher rates of engagement and retention.^19^

For clinical application of remote monitoring with wearable technology to be possible in liver disease, a better understanding of the barriers to adoption and adherence are needed. To address this knowledge gap, we conducted a pragmatic observational multi-center study of wearable observation in patients with cirrhosis, with the primary aim of characterizing data quality and understanding factors which contribute to adherence and drop out. We hypothesized that older patients with more advanced liver disease, less education, and lower experience with wearable technology would have reduced daily adherence.

## Methods

### Study Cohort

Ambulatory patients with cirrhosis were recruited from the Center for Liver Disease and Transplantation at Weill Cornell Medical Center and Columbia University Medical Center over a 24-month period (8/1/2023–8/1/2025). To be eligible, patients were required to be adults, possess a Bluetooth-capable smart phone or tablet and be able to read and provide informed consent in English or Spanish. Cirrhosis was diagnosed clinically through a combination of laboratory, pathology and radiologic factors. Exclusion criteria included a prior history of liver transplantation, transjugular intrahepatic portosystemic shunt, and non-cirrhotic portal hypertension. After providing informed consent, participants underwent a series of surveys, including the Pittsburgh Sleep Quality Index (PSQI)^20^ and the Chronic Liver Disease Questionnaire (CLDQ).^21^ Baseline clinical, sociodemographic, and laboratory data were collected including etiology of liver disease, prior decompensation^22^ (defined by overt HE, ascites or variceal hemorrhage), medication use, and alcohol use. Cognitive status was assessed with the Psychometric Hepatic Encephalopathy Test (PHES),^23^ Stroop EncephalAPP^24^ and Animal Naming Test,^25^ with PHES ≤ −4 in the absence of other chronic neurodegenerative process considered *impaired cognition*. All study activities were approved by the Institutional Review Boards of Weill Cornell Medicine (22–07025079) and Columbia University Medical Center (protocol number: AAAV1203). All study procedures were conducted in accordance with the ethical principles of the *Declaration of Helsinki* and the *Declaration of Istanbul*. De-identified data were transferred between institutions for analytical purposes following execution of an appropriate data use agreement.

### Wearable Data Collection

Participants were given an Oura 3.0 finger-worn fitness tracker for use during the duration of the study and asked to wear the ring day and night. At the beginning of study, approximately 10 participants received the previous generation Oura 2.0 Ring. While the data interpretation algorithm was locked, such as for calculating step counts, and therefore identical between generations, sensor quality and therefore data quality may be higher in patients who received the Oura 3.0. Patients were provided access to data collected from the Oura ring for their personal use, if desired. No other study incentives were provided. Participants were assisted in downloading the application as well as in choosing an appropriately sized ring and given instruction on appropriate wear, preferably on their non-dominant hand, and on how to charge the ring. Participants were provided encoded usernames and email addresses to comply with data security requirements. They were instructed to open the application at least weekly to permit passive data transfer. All data was transferred via Bluetooth to their personal smart device, and to a cloud-based server (“Oura Teams”) where it could be accessed by the study team. If a participant was noted to not be transmitting sleep and activity data one week after enrollment, a single phone call and troubleshooting virtual visit was offered. Additional troubleshooting was available upon request from the participant; a single replacement ring was offered in the case of loss or damage. Death or receipt of liver transplantation were considered competing risk for non-adherence, with data collection halted at that time.

### The Oura Ring and Variables of Interest

The Oura Ring fitness tracker is a consumer grade wearable device that is worn on a finger. It is equipped with several sensors including photoplethysmography (PPG), which utilizes light to assess blood vessel volume across time, a triaxial accelerometer to assess movement and a temperature sensor to assess skin temperature. These signals are then pre-processed using a machine learning model into derived outputs which are available to the research team, including heart rate, respiratory rate, heart rate variability, temperature deviation, sleep staging and metabolic workload on an epoch level. The metabolic equivalent of task (MET) is reported across a 5-minute epoch, where “0” denotes that the participant was not wearing the ring. A total 12 × 12 = 144 epochs are available each day. In our study, a “0” was considered non-wear or non-adherence, such that 6/144 epochs of “0” MET would be considered 5% non-wear, or 95% adherence. A 24-hour day with *no* data available could be related to any of several factors including (1) total non-wear of the device (2) wearing of an uncharged device (3) failure to open the Oura application on their smart device to transmit study data or (4) failure of that device to have internet connection to transmit to the study team. As the ring has internal storage of up to 30 days of data, Bluetooth syncing could allow transfer of previously collected data. In any case, *absence* of received data through the Oura Teams portal was considered non-adherence unless the participant directly withdrew from the study as described above.

### Assessment of Adherence

Feasibility of wearable longitudinal monitoring in cirrhosis was assessed across several measures. As there are currently no standardized metrics for reporting engagement in digital monitoring studies,^26^ adherence measures were chosen prior to analysis based on previous studies. First, the primary measure of overall adherence was *days of full adherence across the 180*-*day study period*. Daily full adherence was defined as ≤ 20% non-wear time over a given 24-hour day. Weekly adherence was defined as 4 + days of full daily adherence in each successive 7-day window after initiation of study data collection. The 80% threshold for full adherence was chosen after an initial sensitivity analysis to determine the wear time needed to meaningfully collect a full day of activity measurement; median recorded steps were not meaningfully different based on 80% vs 100% wear time (median paired difference by subject = 38 steps/day; IQR 663 steps), suggesting that small periods of non-wear were likely during periods of rest such as charging the ring or bathing. Missing data were not imputed in this study.

To study adherence, we encoded a binary vector for each participant indicating whether they were adherent (nonwear time was ≤ 20%) or not on each of the 180 potential study days. This vector was plotted as a heatmap with participants sorted in descending order of number of adherent days. A second vector, encoded with the number of adherent days (0–7) in each of the 25 seven-day weeks, was used to plot weekly adherence trends over time. We used Kaplan-Meier survival analysis to assess participant attrition.^27^ Duration in study was calculated from the first day of data collection to the last day data was received up to a maximum of 180 days. Participants who withdrew from the study were treated as censored at the time of their withdrawal. We used the p-value from a log-rank test to assess significant difference between two Kaplan-Meier curves.

Secondary outcomes of interest included assessment of *frequency and length of data gaps*, which were defined as a 7-consecutive day, or longer, period without a fully adherent day. Frequency and length of data gaps were analysed by encoding a binary vector indicating whether the Oura ring had a wear time greater than 0 each day (i.e. whether the ring was worn for any amount of time each day). Percent of days with wear was calculated for each participant based on their number of eligible days of wear and this was used to categorize participants by engagement. Additionally, we defined *loss to follow up* as termination of data collection and transmission to the study team within 6 months of initiating the study and without voluntarily withdrawing from the study. Finally, *attrition* was defined by calculating the rate of *loss to follow up* across the cohort from day 0 after enrollment to day 180, with censorship from those who were withdrawn from the study, either voluntarily or after receiving liver transplantation or death.

### Subgroups of Interest

To better characterize the association between clinical and sociodemographic status and adherence in liver disease, the probability of adherence was assessed in univariable random intercept logistic regression for several factors. Sociodemographic factors of interest included age, gender, education, past utilization of a wearable fitness tracker, and race (non-Hispanic White or all other). To assess the association of demographic and clinical variables with daily adherence, we used the per patient binary vector indicating whether each day was adherent or not as the outcome in generalized linear mixed models with a random intercept. Models for adherence along clinical variables were run with one clinical variable at a time and adjusted for the following demographic variables: age (continuous, centered); sex (male / female); race/ethnicity (Non-Hispanic White / Other); education (≤ high school / post-secondary education); prior use of fitness tracker (yes / no). An additional model was run with the addition of a categorical variable indicating whether participants had *impaired cognition* by baseline PHES and/or had prior overt HE, defined as a previous hospitalization for overt encephalopathy and/or use of lactulose or rifaximin at enrollment.

#### Stratification by Level of Engagement

After defining adherence across time scales of interest, participants were further stratified according to overall adherence and categorized as having *High, Moderate, or Low Engagement. High Engagement* participants had fully adherent days (80% wear time) on at least 80% of eligible days (end of study collection on 8/1/2025, study withdrawal, or 180 days after enrollment, whichever came first). *Moderate Engagement* participants had fully adherent days 40–80% of eligible days, while *Low Engagement* participants had fully adherent days < 40% of eligible days. We used t-tests to compare means and Fisher’s exact tests to compare proportions between engagement category groups. Logistic regression was used to estimate odds ratios for being in the low engagement category, adjusted for the same demographic variables mentioned above. All statistical analyses were performed in R v4.5.0 using the survival^28^ and lme4^29^ packages.

## Results

Between August 1, 2023 and August 1, 2025 119 patients consented to participate. Of these, 111 began data collection and were analyzed for adherence to the protocol. The mean age of participant was 56, and 54 (48.6%) were female (Table 1). There was a relatively high rate of prior use of wearable fitness tracking, 43.2%, and almost half of patients had experienced a previous decompensation event (41.2%). From the 111 participants, 8 withdrew and 46 were lost to follow up before the 180-day study termination. Overall, a total of 13,011/18,626 potential days had any data for analysis, an average of 117.2 per participant.

### Primary Assessment of Adherence

**Figure 1:**
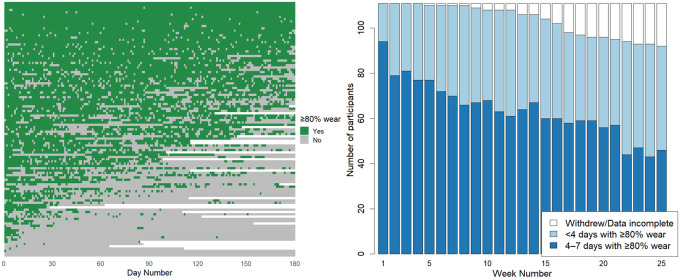
(A) Heatmap showing daily adherence patterns for all participants. An adherent day (green) is one with ≥ 80% wear time. Days with <80% wear time are in gray and white indicates days that were not part of the study either because the participant had withdrawn or because data had not yet been collected. Participants were sorted by descending number of adherent days. (B) Weekly adherence trends across the study period. Stacked bars show the number of participants each week with 4–7 adherent days (dark blue), <4 adherent days (light blue), or who had withdrawn or had incomplete data (white).

Over the study period, and accounting for withdrawals/late enrollment, participants had a median of 65.6% (IQR 24.6% − 82.95%) full adherence on eligible days, defined as non-wear time < 20%. At least one fully adherent week was observed in 102 individuals (94%) of the study cohort. The cohort was more likely to have fully adherent weeks earlier in the study period than later (Fig. 1). Increasing “weeks since start of the study” was associated with a lower odds of having a fully adherent week (OR = 0.89, p < 0.001), based on a mixed-effects logistic regression.

### Data Completeness and Study Dropout

**Figure 2:**
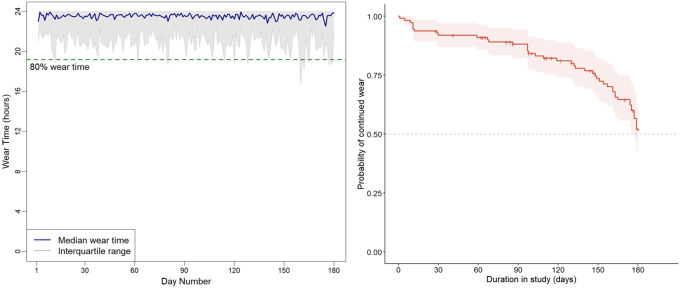
(A) Median wear time across the study period for days the ring was worn (dark blue line). Gray shaded area indicates the 25th – 75th percentile of wear time on those days. (B) Kaplan-Meier survival curve showing the probability of continued wear of the ring over the study period. The shaded area is the 95% confidence interval.

To assess data richness on days in which any data collection occurred, we calculated measured wear time over the course of 24 hours of potential wear time. Across the study period, days with collected data had a median of 26 minutes non-wear time (2% of the day), suggesting that where any data was recorded and transmitted, the likelihood that the day was fully adherent was quite high. This finding remained consistent across the study period (Fig. 2a). Regarding attrition, at 90 days, the probability of remaining in the study was 88%, but by 180 days, 48% of eligible participants had stopped collecting data (Fig. 2b).

### Subgroup Analysis

Across the full study cohort (n = 111), age, race and gender were not significantly associated with the primary outcome (daily wear time > 80%). Likewise, some level of college education or higher and prior use of wearable fitness trackers were not significantly associated with higher adherence. **(Supplementary Table 1)**.

In mixed effects modeling adjusted for age, sex, race, education and prior use of wearable trackers, patients with more advanced liver disease by Child-Turcotte-Pugh (CTP) class (B/C vs A) at time of enrollment had lower adherence (OR 0.29 [95% CI 0.12, 0.68]. Likewise, patients with a history of liver decompensation had lower adherence (OR 0.30 [95% CI 0.12, 0.74]) (Table 2).

Considering factors of progressive liver disease which may be associated with adherence to longitudinal monitoring, both a history of overt HE (OR 0.30 [95% CI 0.13, 0.71]) and impaired cognition (PHES ≤ −4) (OR 0.26 [95% CI 0.10, 0.68]) were strongly associated with reduced adherence. Treated as a continuous variable, increasing PHES score (or improved cognition) was likewise associated with improved adherence (OR 1.22 [95% CI 1.09, 1.37]). Patients who had ascites, defined as use of diuretics or with clinically evident ascites at time of enrollment, had reduced adherence (OR 0.31 [95% CI 0.13, 0.75)], but prior variceal bleeding was not (OR 2.03 [95% CI 0.56, 7.41]) associated with likelihood of adherence. When adjusting for cognitive status, the association with other factors of liver disease severity was attenuated. For example, CTP Class B or C no longer was significantly associated with reduced adherence when adjusting for HE status (OR 0.44 [95% CI 0.17, 1.13]). Considering combinations of cognitive status by PHES and overt HE history, those with both prior overt HE and persistent impaired cognition on enrollment had the lowest adherence, OR 0.17 [95% CI 0.06, 0.47]) to wearable monitoring **(Supplementary Table 2)**.

**Figure 3:**
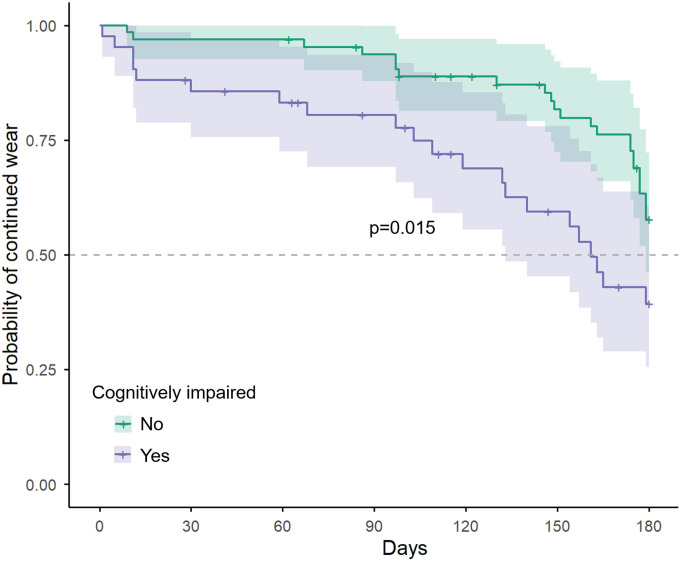
Kaplan-Meier survival curve showing the difference in probability of continued wear of the ring (p=0.015) between those who were cognitively impaired at enrolment (purple) and those who were not (green). Shaded areas represent 95% confidence intervals.

Participants with a history of overt encephalopathy or impaired cognition at enrollment, regardless of prior HE status, also had higher rates of dropout over the study period. Over the first 60 days, for those with signs of cognitive impairment 63% of days were adherent days compared to 72% adherent days for the cognitively unimpaired. Maintained data collection, or lack of dropout, by 180 days was lower among those with cognitive impairment at baseline (Fig. 3).

### Predictive Factors for High and Low Engagement

**Figure 4:**
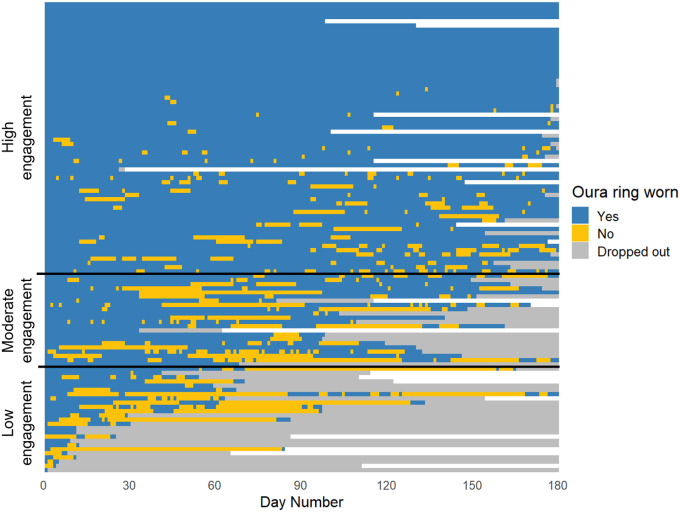
Heatmap showing daily wear patterns of the Oura ring for all participants. Days the ring was worn for any amount of time are blue. Days with no wear are yellow if there was a gap in wear (i.e., occurred between two days with wear) or gray if the participant had dropped out of the study. White indicates days that were not part of the study either because the participant had withdrawn or because data had not yet been collected. Participants were sorted in descending order by percent of possible days with wear and categorized as high engagement (≥ 80% possible days with wear), moderate engagement (>40% and <80%), or low engagement (≤40%).

Across the entire study period, 64 patients were categorized as *High Engagement*, 21 as *Moderate Engagement*, and 26 as *Low Engagement* (Fig. 4). The low and moderate engagement groups were far more likely to have data “gaps”, or periods of total non-wear/transmittal of data. Compared to those with *High Engagement*, those with *Moderate/Low Engagement* had higher likelihood of a data gap greater than one week (29/47 participants vs 15/64 for *High Engagement* participants). They were also more likely to drop out or withdraw from the study before completion (39/47 vs 15/64.) Compared to those with *High Engagement*, participants with *Low Engagement* were more frequently female (61.5% vs 46.9%) and were less likely to have previously used a fitness tracker (30.8% vs 51.6%). There was no statistical difference in likelihood of *High* Engagement compared to *Low* or *Moderate* Engagement by age, gender, education, prior fitness tracker use or race/ethnicity. *Low Engagement* participants were also more likely to have advanced liver disease as measured by CTP B or C disease (66.9% vs 39.0%, *p* = 0.002). Impaired cognition at baseline by PHES conferred higher odds of *Low Engagement* when adjusted for age, gender, education, race/ethnicity and prior use of a wearable tracker (OR 4.44 [95% CI 1.64, 13.0] for PHES ≤ −4).

## Discussion

In a prospective observational cohort in patients with cirrhosis, we found moderately high adherence to a wearable tracking strategy using a commercial fitness tracker. Interestingly, engagement was similar across age, gender, and other key sociodemographic determinants such as race and education. However, we found that baseline cognition, measured by a validated score for detecting covert HE, and a history of clinically diagnosed HE, were associated with attrition and reduced adherence. To our knowledge, this is the first longitudinal study to systematically assess factors related to retention in patients with cirrhosis and provides valuable insight into the feasibility of longitudinal monitoring in this population.

First, despite enrolling a relatively sick population with varying levels of experience with technology, education and across the age and sociodemographic landscape, adherence was high. This supports the feasibility of incorporating remote patient monitoring in this population. Patients with compensated cirrhosis, who may benefit from long-term longitudinal monitoring to detect early clinical changes preceding a first decompensation event, demonstrated high engagement. Specifically, 89% of participants with CTP class A disease had moderate or high engagement, compared with only 44% of those with CTP class C. These findings suggest that initiating remote monitoring programs for early detection and prevention in compensated patients may be feasible with minimal additional incentivization.

Second, we noted a significant difference in adherence among those with more severe cognitive impairment, especially those with prior overt hepatic encephalopathy. Future studies are needed to determine the most effective way to engage and retain this subgroup, which might be the most important group for remote monitoring given the subtle cognitive changes which often precede overt HE, and the challenges in treating HE in the ambulatory setting.

Third, we described the nature of data loss and patient attrition in our study, which is helpful to consider in future studies evaluating the use of wearable technology in disease states, including liver disease. Most notably, we found that data quality was almost absolute on days where the device was worn. There are multiple possible applications of digital biomarkers for clinical care of liver disease. One possible application is to gain an understanding of baseline wellbeing, such as activity levels, exertion, or sleep quality. These may require a shorter duration of observation, but a more complete daily set of data. For example, if a day is missing 12 hours of data, it may not fully represent a patient’s activity level or attained sleep, but key biometrics such as heart rate may be preserved and interpretable. In one study of patients using the Oura tracker during the COVID-19 pandemic, healthcare professionals had relatively high overall adherence (87.8%), albeit with a different threshold for adherence, overall nights where the ring was worn. In our study, the level of attrition after 90 days means consideration of long-term outcomes, such as infrequent events like new episodes of hepatic encephalopathy, may require more direct engagement strategies.

There is a clear demand for at home monitoring solutions in liver disease. A previous study assessed interest in home monitoring in cirrhosis and found that among patients with decompensated disease, 85% were interested in communicating with their physician through an application and 65% were interested in receiving application-based communication regarding their liver care.^30^

Gaps in data collection deserve additional consideration. When developing technology-based interventions for clinical use, the acceptability of “gaps” depends on the outcome/intervention being studied. Literature is sparse to this point, but the relevance of gaps may be lower in patients who are compensated, and in whom technology is being used to simply understand a relatively stable behavior pattern, such as sedentary time or sleep duration. However, conditions which rely upon changes from baseline, such as febrile events or heart rate variability, may need direct patient contact to address gaps in data collection.

In conclusion, given that most studies of wearable technology and digital health have been conducted in healthy populations, further research is needed to understand the feasibility of remote monitoring in high-risk populations like those with cirrhosis. In the absence of reference standards,^31^ future advances in digital technology in liver disease will rely strongly on transparent reporting of adherence measures to facilitate improvements and compare real-world effectiveness of interventions. In our study, despite generally high-quality data acquisition at a high level of adherence, there were clear disease-level differentiators for adherence, which may limit current applicability of remote monitoring and require additional strategies to maintain long-term compliance particularly in the most severely ill patients. Given our finding that cognitive impairment/HE influenced adherence, simplification of monitoring protocols, identification and treatment of HE, and engagement of family members may be able to make monitoring strategies more effective. Conversely, the high adherence in compensated populations suggests potential high value for remote monitoring in low-risk patients who nonetheless face health access inequities such as distance to travel for hepatology assessments. Future studies structured around incentivization and targeted reminders may help to broaden reach and effectiveness of digital health centered interventions and monitoring strategies, improving the future care of patients with cirrhosis.

## Supplementary Files

This is a list of supplementary files associated with this preprint. Click to download.

- SuppcontentClinicalandDemographicDeterminantsofAdherencetoDigitalMonitoringinCirrhosisHC3.10.2026.docx

## Figures and Tables

**Table 1.** Baseline Cohort Demographic and Clinical Variables (n = 111). Shown are median and IQR for continuous variables, and number and % of total for categorical variables

	Median/Number	IQR/% of total
Age	56.0	46.5 – 64.5
Sex (Female)	54	48.6%
Education		
Up to high school	26	23.4%
Post-secondary	81	73.0%
*missing*	4	3.6%
Race/Ethnicity		
Non-Hispanic white	63	56.8%
Other	47	42.3%
*missing*	1	0.9%
Prior use of fitness tracker		
Yes	48	43.2%
No	61	55.0%
*missing*	2	1.8%
Prior Hepatic Decompensation		
Decompensated	65	58.6%
Compensated	46	41.4%
*missing*	0	0.0%
Prior overt HE and/or PHES ≤ -4		
Any HE	65	58.6%
No	43	38.7%
*missing*	3	2.7%
Variceal bleeding history		
Yes	14	12.6%
No	95	85.6%
*missing*	2	1.8%
Medication for Ascites or Clinically Present Ascites at Enrollment		
Yes	45	40.5%
No	66	59.5%
*missing*	0	0.0%
Child-Turcotte-Pugh class		
CTP A	58	52.3%
CTP B	37	33.3%
CTP C	16	14.4%
MELD score	9	7 – 12
*missing*	0	0.0%

**Table 2.** Association of clinical factors with daily adherence using a mixed-effects logistic regression. Odds ratios (OR) have been adjusted for age, sex, education, race/ethnicity and prior use of a fitness tracker and represent the change in odds of daily adherence associated with higher values of the clinical factor (higher MELD score and CTP category B/C indicates worse liver disease; CHE PHES indicates presence of HE; higher PHES score indicates better cognitive performance). HE: Hepatic encephalopathy; CTP: Child-Turcotte-Pugh; PHES: Psychometric hepatic encephalopathy score.

Clinical Factor	OR		95% conf. interval	p-value
History of OHE	0.30	0.13	0.71	0.006
History of Ascites	0.37	0.15	0.91	0.031
History of Variceal Bleeding	2.45	0.66	9.07	0.180
Any Prior Decompensation	0.30	0.12	0.74	0.009
MELD score	0.93	0.85	1.03	0.150
CTP category: B/C (vs A)	0.29	0.12	0.68	0.004
Impaired Cognition (PHES ≤ -4)	0.26	0.10	0.68	0.006
PHES score (continuous)	1.22	1.09	1.37	0.001

## Data Availability

The primary data analyzed in this study is restricted according to Institutional Review Board policies. Deidentified and summary data, Metadata, processed variables, and all numerical source data underlying the figures in this paper will be made available at Zenodo upon acceptance of this manuscript. Additional data will be made available upon request and with appropriate data use agreement.

## Abbreviations

CLDQ: Chronic Liver Disease Questionnaire
CTP: Child-Turcotte-Pugh
HE: Hepatic Encephalopathy
MET: Metabolic Equivalent of Task
PHES: Psychometric Hepatic Encephalopathy Test
PPG: photoplethysmography
PSQI: Pittsburgh Sleep Quality Index
